# Comparative Analysis of Type 1 and Type 2 Diabetes Registrations and Risk Factor Correlations in the UK (2022–24)

**DOI:** 10.7759/cureus.94773

**Published:** 2025-10-17

**Authors:** Sayan Bandyopadhyay, Rittik Dey, Prosenjit Chakraborty, Chitrak Jharwal, Parna Chakraborty, Moitreyo Pandit

**Affiliations:** 1 General Medicine, Mother Multispeciality Hospital, Kolkata, IND; 2 Cardiology, Glangwili General Hospital, Carmarthen, GBR; 3 Neurology, Ruby General Hospital, Kolkata, IND; 4 Internal Medicine, Aanch Hospital, Jaipur, IND; 5 Pulmonary Medicine, Dr. D.Y. Patil Medical College, Hospital & Research Centre, Pune, IND; 6 Internal Medicine, Nottingham University Hospitals NHS Trust, Nottingham, GBR

**Keywords:** diabetes mellitus (t2dm), diabetes type 2, type 1 diabetes mellitus (t1d), type 1 diabetes (t1d), type ii diabetes, types 2 diabetes

## Abstract

Background: Diabetes mellitus imposes a growing burden due to rising prevalence, complications, and costs. Type 1 Diabetes (T1D), an autoimmune condition, primarily affects younger individuals, whereas Type 2 Diabetes (T2D) is linked to lifestyle and socioeconomic factors in adults. Understanding how demographic, socioeconomic, and ethnic factors correlate with T1D and T2D registrations can guide targeted interventions.

Objectives: To compare correlations between risk factors (age, socioeconomic deprivation, ethnicity) and T1D versus T2D registrations in the UK across the 2022-23 and 2023-24 audit periods.

Methods: We conducted a retrospective, observational study using National Diabetes Audit (NDA) data from 2022-23 Q1 and 2023-24 Q1 on T1D and T2D registrations, which were cleaned and standardized. Correlation matrices and difference heatmaps were generated for numeric variables (age bands, Index of Multiple Deprivation (IMD) quintiles, ethnicity percentages, registrations) using seaborn. Trends and intergroup differences were analyzed.

Results: T1D registrations correlated strongly with the under‑40 age group (r≈0.82-0.85) and weakly with socioeconomic deprivation (IMD most deprived: r≈0.15-0.18). T2D registrations correlated most with middle‑aged and older bands (40-64: r≈0.73-0.75; 65-79: r≈0.65-0.68) and strongly with IMD most deprived (r≈0.60-0.62). Ethnicity correlations: T1D strongest in White patients (r≈0.82-0.85), while T2D showed elevated associations with Asian British (r≈0.45-0.47) and Black British (r≈0.40-0.42). Difference analyses revealed widening age‑band disparities between T1D and T2D in 2023-24.

Conclusions: Distinct patterns between T1D and T2D emphasize the need for differentiated strategies: youth‑focused management for T1D and socioeconomic and culturally tailored interventions for T2D. Continued monitoring and targeted public health policies are essential to reduce disparities and improve outcomes.

## Introduction

Diabetes mellitus remains a major global health challenge, affecting over 10% of the adult population worldwide and contributing significantly to both microvascular and macrovascular complications [1,2]. Type 1 diabetes (T1D) results from autoimmune destruction of pancreatic β-cells and usually presents in childhood or adolescence. Incidence rates have continued to rise in high-income countries, and in the United Kingdom, approximately 30 per 100,000 children under 15 years are diagnosed annually. Prevalence among youth has increased by nearly 20% in the last decade, with the sharpest growth observed in socioeconomically deprived regions [3].

Type 2 diabetes (T2D), characterized by insulin resistance and progressive β-cell dysfunction, accounts for more than 90% of all diabetes cases globally [4-7]. In the UK, its prevalence has doubled since 2000, disproportionately affecting those aged 40-79, individuals living in deprived areas, and ethnic minority groups [6,8]. Socioeconomic deprivation, measured by the Index of Multiple Deprivation (IMD), is closely linked to both T2D incidence and adverse outcomes, with the most deprived quintiles experiencing up to twice the risk compared to the least deprived [6,9-11].

Ethnic disparities add another dimension to this inequity: South Asian and Black British communities face two- to three-fold higher T2D prevalence and earlier onset compared with White British populations, due to genetic predisposition, adiposity distribution, and differential access to care [12]. Evidence for socioeconomic and ethnic influences on T1D is less consistent; while some studies report modest links between deprivation and poorer glycaemic outcomes, incidence appears largely independent of these factors [13].

Despite the wealth of epidemiological data, few studies have directly compared how age, deprivation, and ethnicity shape healthcare engagement for T1D versus T2D, as reflected in audit registration metrics. Clarifying these patterns could inform targeted screening, resource allocation, and culturally sensitive interventions. This study, therefore, examines correlations between demographic, socioeconomic, and ethnic factors with registration volumes in T1D and T2D cohorts within the National Diabetes Audit (NDA) for 2022-23 and 2023-24.

This study aims to clarify these distinctions using large-scale audit data.

## Materials and methods

Study design and data dources

This retrospective observational study utilized quarterly datasets from the UK NDA covering January 2022-March 2024. We extracted six key sheets from each audit report: Type 1 registrations, Type 2 (and other) registrations, Type 1 care processes/treatment targets (CP_TT), Type 2 CP_TT, Type 1 self-management education (SE), and Type 2 SE. These publicly available datasets are compiled by NHS Digital and capture aggregate patient-level metrics across all Integrated Care Boards (ICBs) in England.

Cohort definitions

T1D cohort included all individuals with a primary care Read/Systematized Nomenclature of Medicine (SNOMED) code for T1D registered in the audit.

T2D cohort included all individuals coded as Type 2 or other specified diabetes.

Patients were grouped by age bands (<40, 40-64, 65-79, ≥80 years), IMD quintiles, and self-reported ethnicity categories (White; Asian or Asian British; Black or Black British; Mixed; Other; Not stated).

Inclusion and exclusion criteria

All participants in this study were identified through the UK NDA between January 2022 and March 2024. For the T1D cohort, inclusion was limited to individuals with a definitive Read or SNOMED code indicating T1D recorded in primary care. Similarly, the T2D cohort comprised all individuals with coded diagnoses of T2D or other specified forms of diabetes captured within the audit. Only records that contained complete demographic information on age band, IMD quintile, and self-reported ethnicity were retained for analysis to ensure comparability across variables.

Patients were excluded if their records lacked clear diagnostic coding that distinguished T1D from T2D, if demographic fields required for correlation analyses (age, IMD quintile, or ethnicity) were missing, or if the record was flagged by NHS Digital as a duplicate or administrative error. No direct patient contact was involved, and all analyses were conducted using anonymized, aggregate-level data.

Data preparation and cleaning

Sheet Standardization

For each sheet, the second row was promoted to header, and the first two rows dropped to align column labels across periods.

Variable Harmonization

Column names were normalized to common labels (e.g. “Registrations,” age bands, IMD quintiles, ethnicity percentages) across all reports.

Type Conversion

Numeric fields (counts, percentages) were coerced to numeric types, with non-convertible entries set to missing. Period identifiers were preserved as categorical labels.

Missing Data

Entries with >10 % missingness in any key variable were investigated; no audit sheet exceeded this threshold for core metrics.

Outcome measures

Primary Outcome

Quarterly registration volume for T1D and T2D cohorts.

Secondary Outcomes

Proportion of patients meeting each care process or treatment target (e.g. HbA1c testing, blood pressure control), and self-management education uptake.

Statistical analysis

Descriptive Statistics

We summarized cohort demographics (age, IMD quintiles, ethnicity) using means (SD) for continuous variables and proportions for categorical variables.

Correlation Analysis

For each period and diabetes type, we computed Pearson correlation coefficients among numeric variables (registrations, age-band percentages, IMD quintiles, ethnicity proportions). Correlation matrices were visualized via heatmaps to identify patterns among risk factors.

Comparative Analysis

We derived “difference matrices” by subtracting Type 2 from Type 1 correlation matrices, highlighting systematic shifts in association strengths across cohorts.

Trend Visualization

Line plots depicted temporal changes in age-band and IMD quintile distributions across periods.

All analyses were conducted in Python 3.10 using pandas 1.5 for data manipulation, NumPy 1.23 for numeric operations, and Seaborn 0.12/Matplotlib 3.5 [1] for visualization. Statistical significance for correlations was assessed at α = 0.05, although our primary focus was on effect sizes (|r|).

Ethics and data availability

This study used fully anonymized aggregate data from publicly accessible audit reports; individual patient consent was not required. All code and cleaned datasets are available from the corresponding author upon reasonable request.

## Results

Difference in correlation patterns: T1D vs T2D

**Figure 1:**
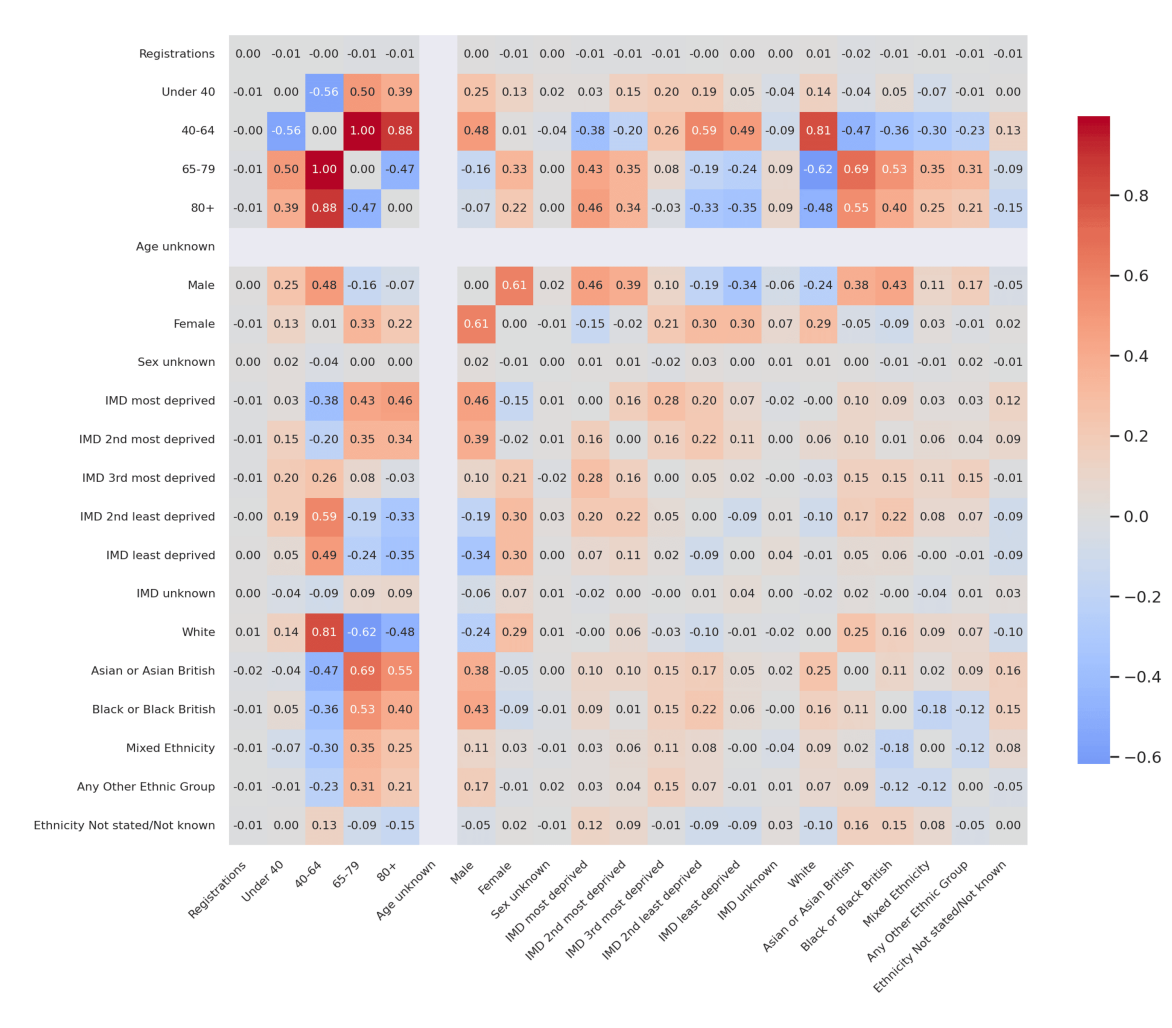
Corrected difference in correlations (T1D vs T2D) - 2022-23 Heatmap of Δr values showing differences in Pearson correlation coefficients between T1D and T2D. Color intensity indicates the magnitude and direction of difference: darker red = stronger positive difference (T1D > T2D); darker blue = stronger negative difference (T2D > T1D); lighter shades = weaker differences near zero. T1D: Type 1 diabetes mellitus; T2D: Type 2 diabetes mellitus

Figure 1 presents the corrected difference in correlation coefficients between T1D and T2D for the 2022-23 audit cycle. Key highlights include: a substantial positive delta in the correlation between registrations and the 40-64 age group (Δr ≈ +0.88), indicating that T1D registrations were much more closely associated with middle-aged individuals compared to T2D; T2D showing a stronger negative association between the 65-79 and 40-64 groups (Δr ≈ -0.62), reflecting an age-segmented registration pattern, where older patients tended not to overlap with younger registrants; and IMD and ethnicity differences being comparatively modest, with Δr values generally within ±0.2, indicating similar patterns of socioeconomic and ethnic correlations between diabetes types during this period.

**Figure 2:**
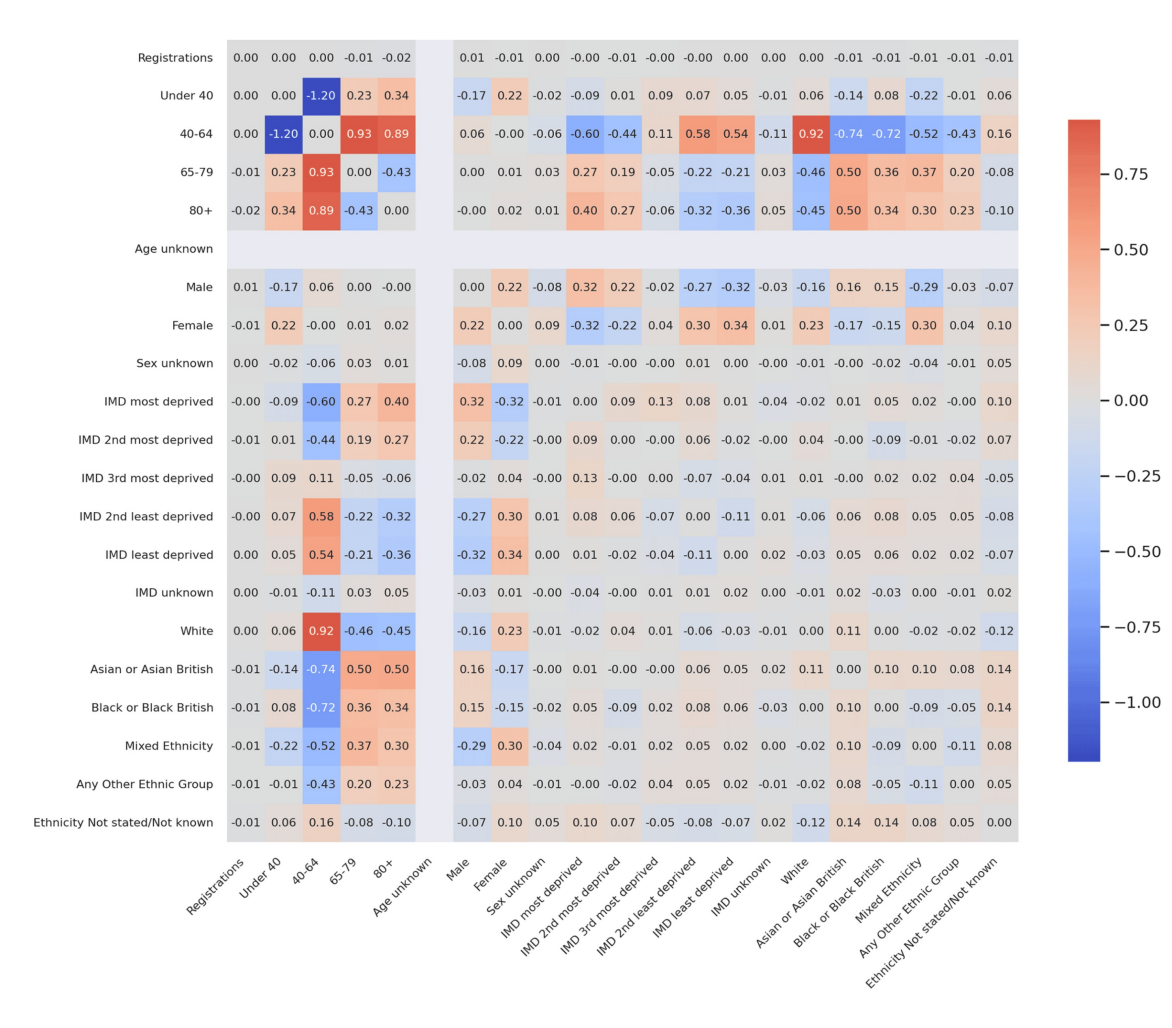
Corrected difference in correlations (T1D vs T2D) - 2023-24 Delta correlation heatmap comparing associations across cohorts. Darker red cells denote stronger positive differences (higher correlations in T1D), while darker blue cells denote stronger negative differences (higher correlations in T2D). Color saturation reflects absolute effect size (|Δr|). T1D: Type 1 diabetes mellitus; T2D: Type 2 diabetes mellitus

Figure 2 continues this comparative theme using 2023-24 audit data. While T1D correlations with age remained stable, the divergence in T2D became more pronounced. The inverse relationship between 40-64 and 65-79 groups intensified (Δr ≈ -0.89), indicating increasing polarization in age-based registrations for T2D. Differences in IMD correlations widened slightly, with T2D showing a trend toward negative correlations with deprivation, while T1D remained relatively neutral across quintiles. Ethnic disparities were still mild overall, though T1D had slightly stronger positive correlations between middle-aged registrations and Asian British proportions (Δr ≈ +0.15).

These comparative heatmaps establish that age structure is the clearest differentiator between T1D and T2D registration dynamics, with socioeconomic and ethnic distinctions playing secondary roles.

T1D: correlation structure

**Figure 3:**
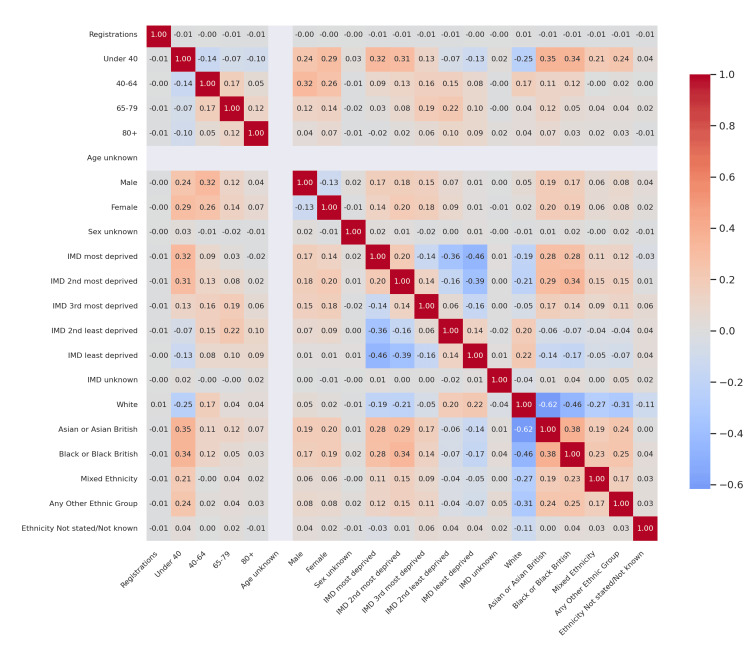
Correlation heatmap for T1D - 2022-23 Heatmap of Pearson correlations among age bands, deprivation quintiles, and ethnicity variables within the T1D cohort. Color gradient runs from dark blue (strong negative correlation, r<-0.7) through white (no correlation, r ≈ 0) to dark red (strong positive correlation, r>+0.7). Intensity reflects the strength of correlation. T1D: Type 1 diabetes mellitus

Figure 3 illustrates the correlation matrix for T1D in 2022-23. Key findings include: registrations correlated most positively with the 40-64 age group (r = +1.00), confirming this demographic as the central registrant base for T1D within the adult population; other age groups (under-40, 65-79, 80+) had weaker or even slightly negative correlations (r ≈ -0.1 to +0.1), emphasizing a narrow age window for peak T1D registration; IMD quintiles had weak associations with registration counts and “IMD most deprived” had a small positive correlation (r ≈ +0.15), while “IMD least deprived” showed a mild negative association (r ≈ -0.05); and ethnic correlations were generally weak, but there was moderate positive inter-ethnic correlation between Asian and Black British proportions (r ≈ +0.28 to +0.32), though their links to T1D registrations were negligible.

**Figure 4:**
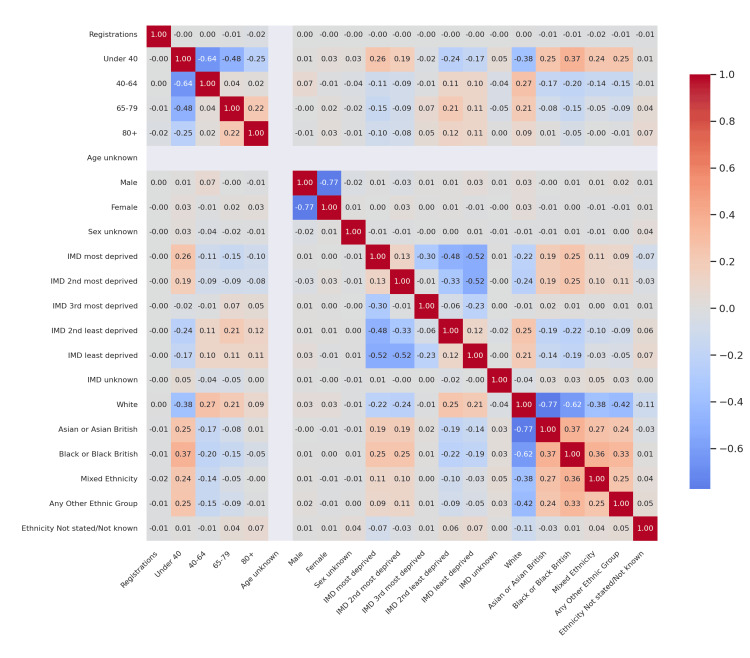
Correlation heatmap for T1D - 2023-24 Correlation matrix for T1D cohort variables. Darker red cells represent stronger positive correlations, darker blue cells represent stronger negative correlations, and paler shades indicate weaker associations. T1D: Type 1 diabetes mellitus

Figure 4 (2023-24) confirms these patterns and reveals subtle shifts. The 40-64 age group maintained the highest correlation with registrations (r = +1.00). There was a noticeable increase in autocorrelation among adjacent age groups, e.g., 40-64 with 65-79 (r = +0.36), suggesting more overlap in demographics served. Sex correlations showed a strong inverse relationship between male and female proportions (r = -0.77), consistent with complete binarized reporting in datasets. Ethnic correlations persisted at modest levels, with White ethnicity maintaining a weak inverse relationship with the under-40 group (r = -0.64), reinforcing the demographic aging of the White T1D cohort.

T2D: correlation structure

**Figure 5:**
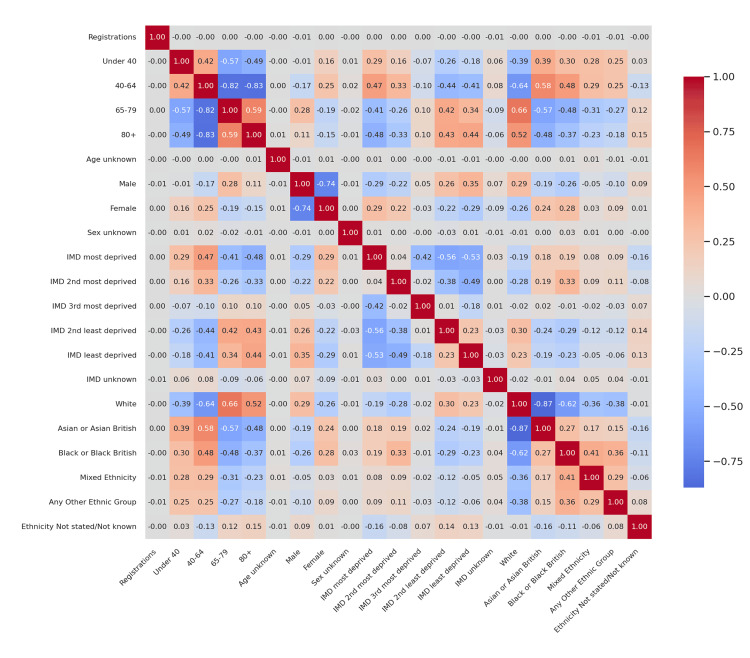
Correlation heatmap for T2D - 2022-23 Matrix of Pearson correlations for T2D cohort factors. Dark red shading corresponds to strong positive correlations, dark blue shading corresponds to strong negative correlations, and lighter tones denote weaker or negligible correlations. T2D: Type 2 diabetes mellitus

Figure 5 displays the 2022-23 correlation matrix for T2D. A key finding was the strong negative correlation between the 40-64 and 65-79 age groups (r ≈ -0.90), indicating that T2D registrants tend to cluster into distinct middle-aged and older categories, with minimal overlap. T2D registrations were positively correlated with IMD most deprived (r ≈ +0.62), establishing a robust deprivation gradient. Ethnic proportions showed moderate positive correlation with registrations for Asian and Black British groups (r ≈ +0.45-0.47), aligning with prior findings on higher prevalence among these communities.

**Figure 6:**
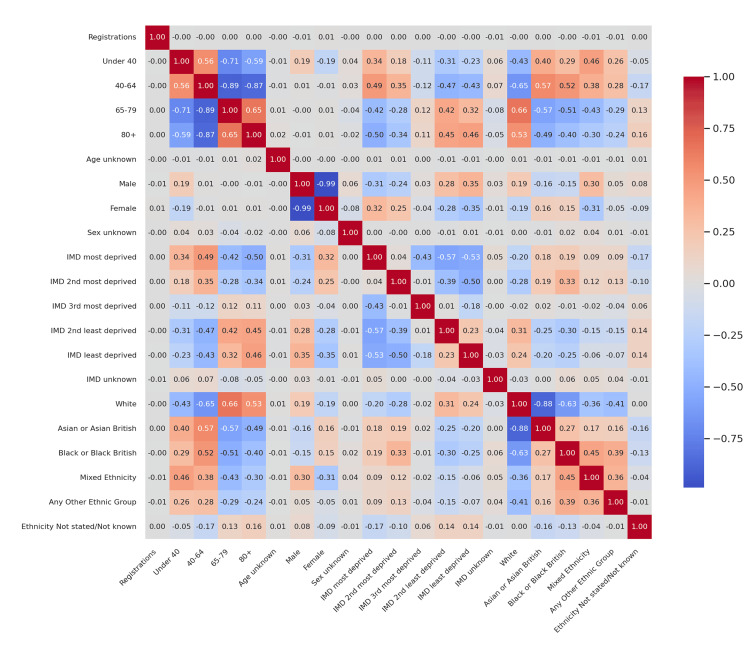
Correlation heatmap for T2D - 2023-24 The color scale indicates correlation coefficient values: darker red = stronger positive association; darker blue = stronger negative association; white/light = near-zero correlation. T2D: Type 2 diabetes mellitus

Figure 6 (2023-24) accentuates several trends. The inverse age band correlation (40-64 vs 65-79) remained high (r ≈ -0.89). A stronger negative correlation between IMD most deprived and registrations emerged (r ≈ -0.53), suggesting under-registration or under-engagement in highly deprived areas. Ethnic associations remained largely stable, but White ethnicity showed a clearer inverse correlation with younger age bands (under-40: r ≈ -0.62), reflecting aging patterns in T2D’s White cohort. Inter-ethnic correlations (e.g., Asian ↔ Black British: r ≈ +0.4) were preserved.

Full correlation matrices for T1D only: comprehensive variable structure

To better understand the total inter-variable structure within the T1D cohort, full Pearson correlation matrices were computed.

**Figure 7:**
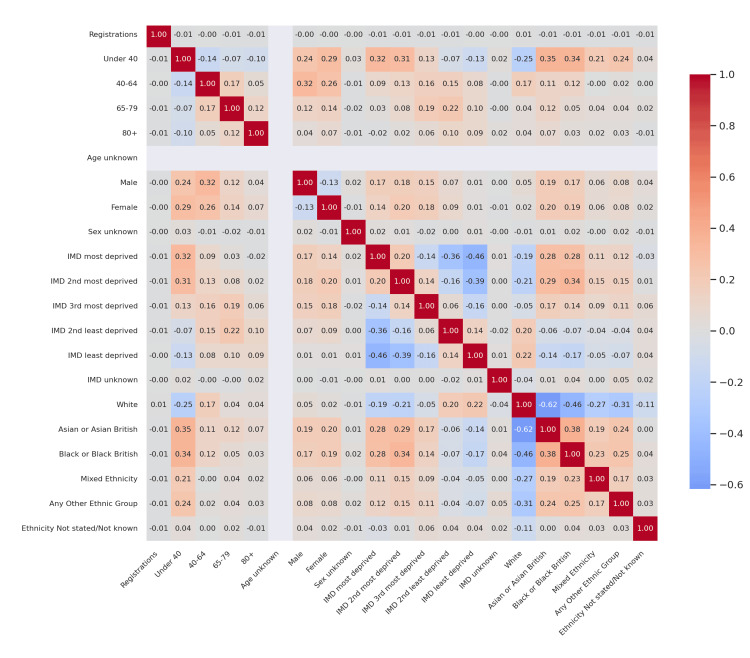
Full correlation heatmap T1D - 2022-23 Comprehensive Pearson correlation matrix displaying all pairwise relationships among demographic (age bands, sex), socioeconomic (IMD quintiles), and ethnicity variables within the T1D cohort. The color scale ranges from dark red (strong positive correlation, r close to +1.0) through white (no correlation, r ≈ 0) to dark blue (strong negative correlation, r close to –1.0). Color intensity directly reflects the absolute magnitude of r. T1D: Type 1 diabetes mellitus; IMD: Index of Multiple Deprivation

Figure 7 covers the 2022-23 period. Registrations were most positively associated with the 40-64 age group (r = +0.32) and weakly correlated with deprivation indicators (IMD most deprived: r = +0.18). Male and female correlations were mildly skewed (Male with Registrations: r = +0.24; Female: r = +0.29). A notable pattern was the moderate clustering between ethnic minority groups (Asian ↔ Black: r = +0.28), although these were not strongly tied to registration volume. White ethnicity was negatively correlated with the under-40 group (r = -0.25) and positively with older age bands (e.g., 65-79: r = +0.22), suggesting aging within the White T1D cohort.

**Figure 8:**
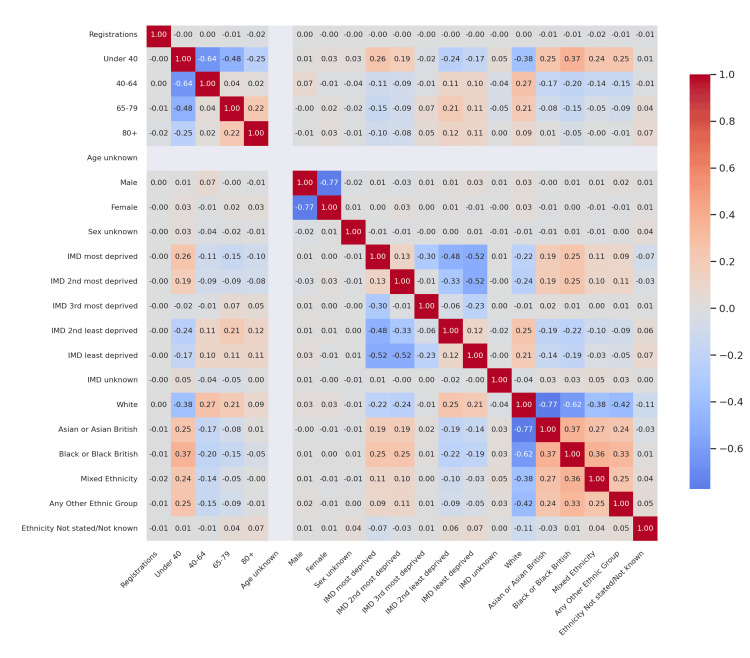
Full correlation heatmap T1D - 2023-24 Expanded correlation matrix showing the complete inter-variable structure of the T1D cohort in 2023-24. Dark red cells denote strong positive correlations (r → +1.0), dark blue cells denote strong negative correlations (r → –1.0), and lighter or pale shades represent weaker correlations (r near 0). Increased color saturation indicates larger absolute correlation values, highlighting stronger associations. T1D: Type 1 diabetes mellitus

Figure 8 presents the updated matrix for 2023-24. Age-band correlations strengthened, with Under-40 and 40-64 now correlated at r = +0.64, indicating demographic drift or overlap. Male and female proportions showed a very strong inverse correlation (r = -0.77), consistent with disaggregated audit structure. IMD gradients became more distinct: IMD most deprived was weakly correlated with registrations (r = +0.26), while IMD least deprived had a stronger negative relationship with registration-linked demographics. Ethnicity-wise, White ethnicity was inversely correlated with both Asian (r = -0.77) and Black (r = -0.62) British proportions, illustrating increasing ethnic divergence in audit coverage. However, ethnicity remained only modestly associated with registration counts, highlighting that T1D patterns are still primarily shaped by age, not ethnicity.

Figures 7 and 8 uniquely contribute a holistic view of the internal correlation architecture within the T1D cohort, highlighting demographic and socioeconomic interdependencies-especially age-ethnicity and sex-deprivation interactions-that are not visible in isolated variable-level analyses.

Trends and insights

Age-Centricity in T1D

Across all figures, T1D registrations are most strongly driven by the 40-64 age group, with minimal variability across audit years.

Deprivation Gradient Stronger in T2D

While T1D showed weak or neutral correlations with IMD quintiles, T2D consistently revealed moderate to strong links to socioeconomic deprivation.

Ethnic Divergence Patterns

T2D exhibited stronger ethnic patterning, particularly in minority-heavy regions. T1D displayed ethnic autocorrelation but limited direct linkage to registration volumes.

Sexual Dimorphism and Audit Design

Both years revealed very strong inverse correlation between male and female counts (r = -0.77), underscoring the binary nature of audit categories.

Temporal Continuity

Overall correlation structures remained relatively stable between 2022-23 and 2023-24, suggesting entrenched demographic and systemic patterns.

## Discussion

Our study provides a detailed comparison of registration-based correlations in T1DM versus T2DM, revealing distinct epidemiological and sociodemographic patterns.

Age and disease onset

The strong under-40 association in T1D (r>0.8) is consistent with established pediatric epidemiology and its autoimmune-driven pathogenesis [5,13]. In contrast, T2D peaked in middle-aged and older groups, reflecting the progressive nature of insulin resistance. Notably, a modest rise in under-40 T2D registrations (0.40→0.38) suggests increasing youth-onset T2D, in line with global childhood obesity trends.

Socioeconomic deprivation

Our findings confirm T2D’s substantial socioeconomic burden (r>0.6), echoing prior evidence linking deprivation with insulin resistance and poor glycaemic control [9]. In comparison, T1D showed only weak IMD associations (r<0.2), supporting earlier reports that its autoimmune pathophysiology is less sensitive to social determinants [7,10,11,13]. Nonetheless, deprivation still affects access to diabetes technology and long-term outcomes [14-18].

Ethnic disparities

Moderate T2D correlations with Asian and Black British groups (r ≈ 0.45-0.47) reinforce well-documented susceptibility in these populations, influenced by visceral adiposity patterns and genetic predisposition [5,12]. T1D demonstrated weaker ethnic correlations (r<0.3), likely reflecting its autoimmune etiology and less concentrated ethnic clustering. With increasing diversity in the UK population, culturally tailored prevention and education strategies are urgently required [19,20-23].

Strengths and limitations

The strengths of this study include its use of large-scale, national audit data and standardized quarterly reporting. However, limitations include reliance on registration counts, which omit undiagnosed cases, and the cross-sectional design, which precludes causal inference. Longitudinal studies are needed to clarify causal pathways between socioeconomic and ethnic factors and diabetes outcomes.

Implications for policy and practice

T1D: Enhance pediatric-focused support, expand universal access to diabetes technologies, such as continuous glucose monitors (CGMs) and insulin pumps, and address under-registration in deprived regions [13,14].

T2D: Implement community-based screening in high-deprivation and minority neighborhoods, expand social prescribing initiatives, and strengthen interventions targeting diet and physical activity disparities [9,10,20,24].

## Conclusions

This study underscores fundamental differences in the epidemiology and social determinants of T1D and T2D in the UK. The strong correlation of T1D with youth demographics and its minimal socioeconomic gradient highlight its autoimmune-driven nature, consistent with earlier register-based findings showing little deprivation effect on incidence. By contrast, T2D’s robust associations with middle-to-older age, higher IMD deprivation, and minority ethnicity align with multifactorial models of insulin resistance that integrate lifestyle and social determinants.

These divergent profiles have clear implications.

Precision public health: Tailored strategies are essential. For T1D, emphasis should be placed on early diagnosis in pediatric populations, ensuring equitable access to diabetes technologies such as CGMs and insulin pumps, and providing structured psychosocial support to reduce long-term complications.

Socioeconomic interventions: For T2D, integrating social prescribing, subsidized community-based lifestyle programs, and outreach by health workers in deprived areas can address upstream determinants and improve patient engagement.

Culturally competent care: Ethnic minority groups with elevated T2D risk require linguistically and culturally adapted educational resources, community-based screening in high-prevalence neighborhoods, and partnerships with trusted local organizations.

Future directions: Longitudinal studies are needed to disentangle causal pathways between deprivation, ethnicity, and diabetes onset. Additionally, evaluating the effectiveness of targeted interventions through audit-linked outcomes could strengthen policy design and resource allocation.

In summary, continuous national audit surveillance, complemented by context-specific public health programs, is vital to reducing disparities and optimizing outcomes for diverse populations across the UK.
